# SOCIAL ISOLATION, LONELINESS, AND CAREGIVER BURDEN AMONG PAID AND UNPAID CAREGIVERS OF HOMEBOUND OLDER ADULTS

**DOI:** 10.1093/geroni/igz038.3505

**Published:** 2019-11-08

**Authors:** Amy Y Sun, Emily Finkelstein, Karin Ouchida

**Affiliations:** 1 Weill Cornell Medicine, New York, New York, United States; 2 Weill Cornell Medicine, New York, United States

## Abstract

Caregivers of homebound older adults may have high levels of burden and more vulnerability to social isolation and loneliness, given that their care recipients are more physically frail and isolated. Existing literature has not fully investigated differences between paid and unpaid caregiver burden or their experiences of social isolation. We interviewed paid (n=21) and unpaid family caregivers (n=22) of homebound older adults in a hospital-affiliated geriatric house call program. We used validated survey instruments to measure social isolation, loneliness, and caregiver burden, and semi-structured interviews to solicit qualitative data. In our sample, 42% of caregivers helped with 5+ ADLs and 58% with 5+ iADLs. Using the Caregiver Burden Inventory, burden types between caregivers were compared with chi-squared tests. Compared to paid caregivers, unpaid family caregivers experienced more “developmental” burden such as “missing out on life” (p<0.01). Paid caregivers exhibited more “time” burden, such as “not having a minute’s break from caregiving responsibilities” (p<0.01). 44% of caregivers were considered socially isolated according to the Berkman-Syme Social Index. However, using the UCLA 3-item Loneliness Scale, few caregivers felt lonely (14%). Thematic analysis revealed that family caregivers desired support groups but time pressures limited their participation (23%). Interestingly, smart phones were regularly cited as a tool for alleviating loneliness for paid caregivers when alone on the job (19%), a novel finding. Findings suggest that caregivers of the chronically ill and physically isolated may be at particular risk of social isolation. Network based social support interventions may mitigate some of these vulnerabilities.

